# Cross-sectional study to evaluate burnout among pharmacy staff in Saudi Arabia during COVID-19 pandemic

**DOI:** 10.1016/j.jsps.2022.01.017

**Published:** 2022-01-31

**Authors:** Lobna A. Aljuffali, Munerah O. Alshabanah, Haya M. Almalag

**Affiliations:** a Department of Clinical Pharmacy, King Saud University, College of Pharmacy, Riyadh, Saudi Arabia; b King Saud University, College of Pharmacy, Riyadh, Saudi Arabia

**Keywords:** Burnout, Pharmacists, Pandemics, COVID-19, Professional, Caregivers

## Abstract

**Background:**

Coronavirus disease 2019 (COVID-19) has placed healthcare workers, including pharmacists, at an increased risk of infection and has increased their workload, which could lead to burnout. Therefore, the present study aimed to measure the levels of burnout among hospital and community pharmacists in Saudi Arabia during the COVID-19 pandemic and to identify the risk factors associated with burnout.

**Methods:**

In the present cross-sectional study, an online survey was distributed among community and hospital (inpatient, outpatient, clinical, and administration) pharmacists in Saudi Arabia. The Copenhagen Burnout Inventory (CBI) survey was used to measure burnout levels. This 19-item survey covered three aspects: personal, work, and patient-related burnout. The survey included questions about socio-demographic factors and the major causes of psychological distress among pharmacists during the COVID-19 pandemic. Inferential statistics were used for data analysis.

**Results:**

A total of 502 pharmacists were included in the present study. Of these, 59.1% were categorized as having clinically relevant burnout levels (scores ≥ 50).

Univariate analysis revealed that the burnout level was significantly higher (p < 0.05) among pharmacists who were younger, were females, had lesser years of experience, or worked in the community pharmacy. The main factors associated with high burnout levels among community pharmacists were younger age, lesser years of experience, male gender, non-Saudi nationality, and higher numbers of customers. In the COVID-19 part of the survey, burnout was found to be strongly related to the COVID-19 pandemic. Fears of getting infected or of a family member getting infected, fears of the pandemic lasting for too long, and increased working hours were factors contributing to increased burnout levels during the COVID-19 pandemic with unadjusted β of 8.336 and (95% CI 7.082–9.589, p value < 0.001). A lack of supportive work culture and sleeping disturbances were also found to be related to high burnout levels (p < 0.001).

**Conclusions:**

Pharmacy staff in Saudi Arabia were found to experience high levels of burnout. The major risk factors causing burnout included younger age, female gender, lesser years of experience, a lack of supportive work culture, sleep disturbances, worries associated with increased workload during the pandemic, fears of getting infected, and increased working hours.

## 1 Introduction

In March 2020, the coronavirus disease 2019 (COVID-19) outbreak was declared a pandemic by the World Health Organization WHO (WHO, 2020). Healthcare professionals from different disciplines are working together to face this pandemic and provide care to patients. As a result of the pandemic, healthcare workers are under immense strain because of increased workload and fears of getting infected and thereby infecting family members (Khasne et al., 2020). According to the (WHO), burnout is a syndrome conceptualized as resulting from chronic workplace stress that has not been successfully managed. It is characterized by three dimensions: feelings of energy depletion or exhaustion, increased mental distance from one’s job or feelings of negativism or cynicism related to one’s job, and reduced professional efficacy (WHO, 2019).

Studies that been held in Italy and Spain on healthcare providers, including pharmacists, have revealed a high prevalence of burnout during the COVID-19 pandemic (Barello et al. 2020), (Di Monte et al., 2020), (Martínez-López et al., 2020). A study among community pharmacists in France found that up to 35% of pharmacists reported psychological disturbances, including burnout (Lange et al., 2020). Another study in the USA reported that burnout affected more than half of healthcare pharmacists (Jones et al., 2021).

Recent studies before the COVID-19 pandemic in France, USA and Japan have assessed burnout among pharmacists practicing in hospital settings and community pharmacies and reported a high prevalence of burnout syndrome (Balayssac et al., 2017), (Higuchi et al., 2016), (Jones et al., 2017). Studies in central Italy and North Carolina, USA have reported that pharmacists are at risk of burnout (Kang et al., 2020), (Protano et al., 2019). Burnout is associated with several factors, including anxiety and the consumption of health resources (Balayssac et al., 2017). Certain factors, such as female gender, working in a primary distribution role, and working for long hours per week, may be associated with higher burnout levels (Kang et al., 2020).

On February 15, 2020, a study on the prevalence of burnout among hospital pharmacists at National Guard Hospital in Riyadh, Saudi Arabia, was published. In that study, burnout was detected in a quarter of the pharmacists working at the hospital (Alharbi et al., 2020). However, in Saudi Arabia, no study has assessed burnout levels among pharmacists under the stress of the COVID-19 pandemic. Moreover, very limited studies have assessed the prevalence of burnout syndrome among pharmacists worldwide. Hence, the present study aimed to assess the prevalence of burnout syndrome among hospital and community pharmacists in Saudi Arabia during the COVID-19 pandemic. As few studies have assessed risk factors associated with burnout in pharmacists or explored the reasons for burnout through qualitative analysis, we also aimed to identify the risk factors associated with burnout among hospital and community pharmacists in Saudi Arabia during this pandemic.

## 2 Methods

### 2.1 Study design, study population, and data tools and instruments

In the present cross-sectional study, an online survey was distributed among community and hospital pharmacists in Saudi Arabia between June 19 and September 30, 2020. The study also involved a brief qualitative assessment. The study was conducted and reported in accordance with the Strengthening the Reporting of Observational Studies in Epidemiology (STROBE) (von Elm et al., 2007) checklist for the cross-sectional part.

The survey consisted of three parts. The first part included a validated survey, the Copenhagen Burnout Inventory (CBI), consisting of 19 questions that covered cover three aspects: personal burnout (six items), work-related burnout (seven items), and patient-related burnout (six items) (Kristensen et al., 2005, Nfa.dk CBI “English version”, 2021, Nfa.dk CBI, 2021).

The second part consisted of questions related to the major causes of psychological distress among pharmacists during the COVID-19 pandemic, such as feeling more burnout during the pandemic, long working hours, sleep disturbances, fears of the pandemic going on for too long, the risk of getting infected or of a family member getting infected, and a lack of supportive culture at the workplace.

The third part consisted of sociodemographic and job-related questions. Responses were scored using a five-point Likert scale. At the end of the survey, an open-ended question was added to assess the respondents’ opinions and experiences of working during the pandemic (Appendix A).

The survey was generated using Google Forms. To ensure content and face validity, the survey was pilot tested. A total of 25 pharmacists were invited to complete the pilot questionnaire, and a structured feedback form was provided to obtain their comments about the questionnaire design and layout, the clarity of information, the time of completion, and their objections (if any) regarding answering any question. The questions were revised and modified according to the feedback received.

All pharmacists and pharmacy technicians who were involved in direct patient care and worked in community and hospital pharmacies, including inpatient, outpatient, clinical, and administration domains, were included in the study. Students, academics, pharmacists working in drug companies, manufacturers, and Saudi Food and Drug Administration pharmacists were excluded because they lacked direct patient involvement. To enable the recruitment of pharmacists from all parts of Saudi Arabia, different social media networks were used, namely Twitter® WhatsApp® and Telegram®. In addition, pharmacists listed in the Saudi Pharmaceutical Society were emailed.

### 2.2 Statistical analysis

Data were coded and entered into the Statistical Package for the Social Sciences (SPSS), version 26. Normally distributed data are presented as the means and standard deviations (SDs), while categorical data are presented as the numbers and percentages. The total burnout score was calculated using a 0- to 100-point scale. Respondents with a mean score ≥ 50 were classified as experiencing burnout. Scores of 50–74 were considered moderate burnout, scores of 75–99 were considered high burnout, and a score of 100 was considered severe burnout. In addition, the responses (n, %) and average scores were separately calculated for each question. The association of baseline demographics and work-related characteristics with burnout scores was assessed using the *t*-test or analysis of variance (ANOVA) in order to compare the means of normally distributed data. Pearson’s correlation co-efficient was used to link burnout levels with other continuous variables, of which at least one followed a normal distribution. A radar chart was used to display the most influential question with regard to the burnout score. Multiple linear regression was used to determine the relationship between the primary outcome variable (burnout score-dependent variable) and different demographics, work-related characteristics, and COVID-19-related findings, taking into consideration age and gender as confounding variables. The internal consistency of the surveys was assessed using Cronbach’s alpha test for reliability. For the qualitative part, thematic analysis was performed on free text responses.

### 2.3 Ethical approval.

Ethics approval for the present study was obtained from the Ethics Committee/Institutional Review Board of Health Sciences Colleges Research on Human Subjects, King Saud University College of Medicine, on May 5, 2020 (project no. E-20–4851). The identity of the participants was not disclosed for privacy, and the completion of the questionnaire implied their consent.

## 3 Results

### 3.1 Demographic characteristics, work-related factors, and burnout

Out of 2000 pharmacists who received our survey, 520 pharmacists responded to our survey (response rate, 26%); 18 of these were excluded because they did not match our inclusion criteria. Thus, 502 pharmacists were included in the present study. Their mean (±SD) age was 31 (±8) years. Most respondents were female (51.8%). Moreover, most respondents were Saudi pharmacists (88.9%). Approximately 59.7% of the respondents were single, divorced, or widowed. Furthermore, approximately 38.3% of the respondents had a bachelor’s degree, while 23.8% had a PharmD degree. Of the 502 respondents, 32.7% had 1–5 years of experience. The average working hours of most respondents before and during the pandemic were 21–40 h per week. Most respondents identified themselves as community pharmacists (24%), followed by inpatient pharmacists (22.8%) and clinical pharmacists (21.8%). With regard to their lifestyles, more than 80% of the respondents did not smoke. The mean (±SD) number of patients seen per day was 2 (±2). In addition, in the case of most hospital pharmacists, the number of beds in the hospital ranged from 101 to 400. With regard to the three dimensions of burnout, the highest score was found for personal burnout (67.3%). In addition, approximately 59.1% of the respondents could be categorized as having clinically relevant burnout levels (scores ≥ 50).

In terms of the respondents’ demographics, the highest burnout scores were observed in community pharmacists (scores, 55.7), pharmacists with a diploma degree (58), single pharmacists (54.4), smokers (56.8), pharmacists with experience between 6 and 10 years (58), and pharmacists with > 80 weekly working hours before or during the pandemic (burnout scores before the pandemic, 62.7; burnout scores during the pandemic, 59). Univariate analysis revealed that younger age (p < 0.001), female gender (p = 0.022), lesser years of experience (p < 0.001), and working in the community pharmacy (p < 0.015) were significantly associated with burnout. Assessment of the demographics of community pharmacists revealed that younger age (p < 0.001), male gender (p = 0.01), non-Saudi nationality (p < 0.001), higher numbers of costumers (p = 0.087), and lesser years of experience (p < 0.001) were the main factors associated with high burnout levels. The baseline demographics and burnout levels are listed in Table 1 along with resulting the p values obtained from univariate analysis.

**Table 1:** Baseline demographical characteristics with resulting p value of difference using univariate analysis Pearson correlation *t* test or analysis of variance whenever appropriate.

**Factors**	**Categories**	**Number (%)**	**Burnout Score mean (SD)**	**P value**
**Age in years mean (Standard Deviation)**		31 (8)	Coefficient −0.144	0.001*
**Burnout score mean (SD)**			53.7 (18.8)	
**Burnout ≥ 50**	Total (%)	298 (59.1)		
	**Personal (%)**	**339 (67.3)**		
	Work-related (%)	330 (65.5)		
	Patient related (%)	226 (51.8)		

**Pharmacy related occupation**	Pharmacy technician	15 (3.0)	55.0 (24.7)	0.015*
	**Community pharmacist**	**121 (24.0)**	**55.7 (18.9)**	
	Pharmacist working in Out-Patient hospital pharmacy	84 (16.7)	55.3 (19.3)	
	Pharmacist working in In-Patient hospital pharmacy	115 (22.8)	55.6 (16.9)	
	Pharmacist working in administration	59 (11.7)	45.9 (19.0)	
	Clinical pharmacist	110 (21.8)	52.2 (18.2)	

**Gender**	Male	243 (48.2)	51.7 (19.8)	0.022*
	**Female**	**261 (51.8)**	**55.5 (17.5)**	

**Highest academic degree**	**Diploma**	**22 (4.4)**	**58.0 (23.7)**	0.884
	BSc Pharmacy	193 (38.3)	53.9 (17.9)	
	Pharm D as first degree	120 (23.8)	54.1 (17.3)	
	PGY1	28 (5.6)	52.8 (19.0)	
	PGY2	33 (6.5)	55.4 (19.0)	
	Pharm D post BSc	16 (3.2)	52.9 (16.7)	
	MSc pharmacy	54 (10.7)	51.5 (23.1)	
	PhD	38 (7.5)	51.1 (18.9)	

**Marital status**	**Single-divorced-widowed**	**301 (59.7)**	**54.4 (17.3)**	0.290
	Married	203 (40.3)	52.6 (20.7)	

**Nationality**	Saudi	448 (88.9)	53.9 (18.5)	0.420
	Non-Saudi	56 (11.1)	51.8 (21.1)	

**Smoking**	No	431 (85.5)	53.1 (18.2)	0.126
	**Yes**	**73 (14.5)**	**56.8 (21.5)**	

**Years of experience**	<1 Year	131 (26.0)	50.5 (18.2)	<0.001*
	1–5 Years	165 (32.7)	57.7 (15.9)	
	**6**–**10 Years**	**86 (17.1)**	**58.0 (18.2)**	
	11–15 Years	57 (11.3)	53.1 (22.4)	
	>15 Years	65 (12.9)	44.4 (19.7)	

**Weekly working hours before the pandemic**	21–40	270 (53.6)	54.0 (17.9)	0.160
	41–60	206 (40.9)	52.3 (19.2)	
	61–80	22 (4.4)	60.0 (21.5)	
	**>80**	**6 (1.2)**	**62.7 (27.8)**	

**Weekly working hours after the pandemic (in the last two months)**	21–40	250 (49.6)	53.6 (17.6)	0.072
	41–60	175 (34.7)	51.7 (19.2)	
	61–80	50 (9.9)	57.9 (20.5)	
	**>80**	**29 (5.8)**	**59.0 (21.0)**	

**Number of patients seen per day mean (SD)**	2 (2)		Coefficient 0.008	0.841

**Number of beds in the hospital**	NA	172 (34.1)	55.0 (19.3)	0.223
	<100	49 (9.7)	49.6 (15.9)	
	101–400	112 (22.2)	54.1 (17.7)	
	401–800	51 (10.1)	49.0 (18.9)	
	**801**–**1000**	**44 (8.7)**	**55.1 (18.0)**	
	>1000	76 (15.1)	54.8 (20.6)	

* According to significant value of ≤0.05; **Bold** indicates highest burnout score; SD: standard deviation.

**Fig. 1:**
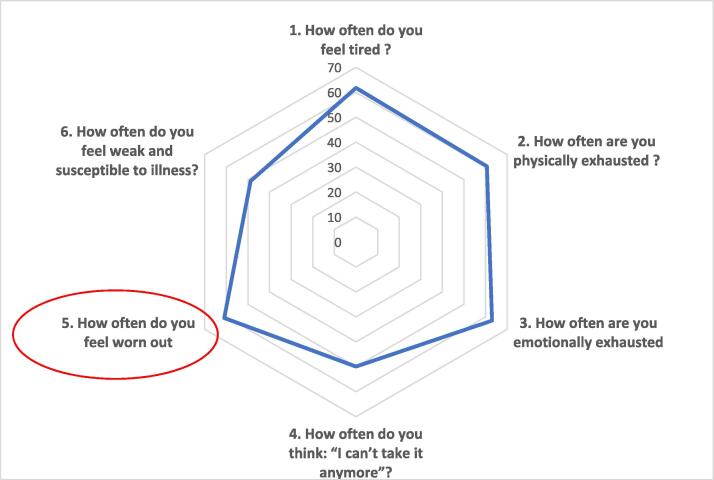
Radar chart of personal-related burnout questions with most influencing question.

**Fig. 2:**
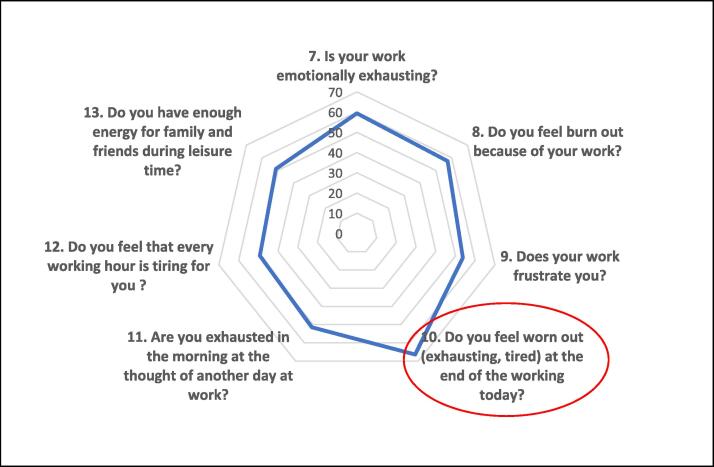
Radar chart of work-related burnout with most influencing question.

**Fig. 3:**
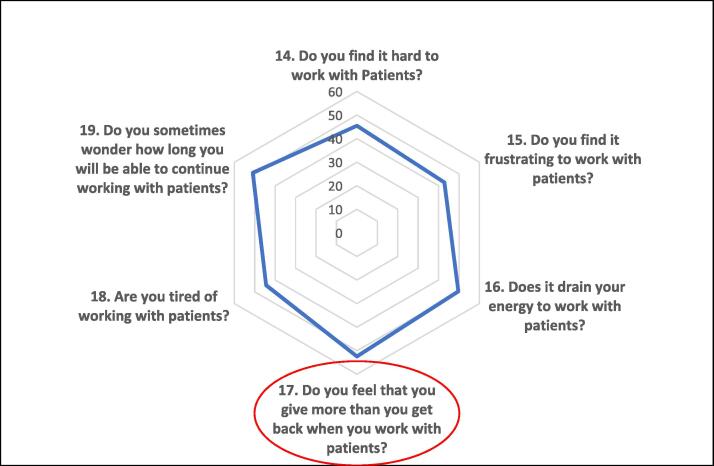
Radar chart of patient-related burnout with most influencing question.

When exploring personal, work-related, and patient-related factors using radar charts (Fig. 1, Fig. 2, Fig. 3, respectively), general questions, such as those pertaining to feeling tired (personal), feeling worn out (work-related), and feeling underappreciated (patient-related), had the maximum effect on directing the pharmacists’ responses and assessing their burnout levels.

### 3.2 COVID-19 survey and burnout levels

Most respondents agreed that they experienced burnout during the COVID-19 pandemic; the more they agreed, the greater was their burnout score on univariate analysis (p < 0.001). A lack of supportive work culture also significantly increased burnout levels (p < 0.001). Moreover, having too little sleep or insomnia, waking up at night, or sleeping late on weekdays was associated with higher burnout scores (p < 0.001). The questions related to COVID-19 and its relation to burnout score are listed in Table 2.

**Table 2:** Questions related to COVID 19 and its relation to burnout score analysed using univariate analysis *t* test or analysis of variance.

		Number (%)	Mean (SD)	P value
1. I feel more burnout now as compared to before the crisis of COVID	Strongly disagree	27 (5.4)	31.9 (19.8)	<0.001*
	Disagree	50 (9.9)	38.3 (19.7)	
	Neither agree or disagree	96 (19.0)	49.9 (15.3)	
	Agree	188 (37.3)	53.6 (14.0)	
	**Strongly agree**	**143 (28.4)**	**65.8 (17.2)**	

2. I am worried about becoming infected	Strongly disagree	24 (4.8)	38.8 (20.6)	<0.001*
	Disagree	45 (8.9)	44.6 (17.6)	
	Neither agree or disagree	86 (17.1)	47.4 (17.8)	
	Agree	172 (34.1)	51.2 (14.6)	
	**Strongly agree**	**177 (35.1)**	**63.5 (18.4)**	

3. I am worried about my family becoming infected	Strongly disagree	7 (1.4)	30.1 (18.0)	<0.001*
	Disagree	15 (3.0)	47.0 (16.1)	
	Neither agree or disagree	52 (10.3)	45.3 (16.8)	
	Agree	128 (25.4)	48.6 (17.6)	
	**Strongly agree**	**302 (59.9)**	**58.1 (18.3)**	

4. I am worried about this going for too long	Strongly disagree	12 (2.4)	43.2 (25.8)	<0.001*
	Disagree	25 (5.0)	45.8 (18.9)	
	Neither agree or disagree	86 (17.1)	46.3 (16.7)	
	Agree	160 (31.7)	49.9 (16.6)	
	**Strongly agree**	**221 (43.8)**	**60.8 (18.2)**	

5. My workplace shows a Supportive culture	**Never**	**45 (8.9)**	**65.6 (21.5)**	<0.001*
	Rarely	68 (13.5)	58.7 (17.6)	
	sometimes	146 (29.0)	51.8 (13.3)	
	Often	156 (31.0)	50.3 (17.6)	
	Always	89 (17.7)	52.8 (24.2)	

6. I get too little sleep at night	Never	22 (4.4)	34.0 (16.7)	<0.001*
	Rarely	73 (14.5)	44.8 (17.6)	
	sometimes	175 (34.7)	49.1 (15.5)	
	Often	125 (24.8)	57.5 (16.3)	
	**Always**	**109 (21.6)**	**66.6 (18.4)**	

7. I have insomnia	Never	76 (15.1)	38.9 (16.5)	<0.001*
	Rarely	108 (21.4)	47.8 (15.4)	
	sometimes	147 (29.2)	51.8 (14.9)	
	Often	98 (19.4)	61.4 (16.2)	
	**Always**	**75 (14.9)**	**70.8 (18.2)**	

8. I wake up often during the night	Never	59 (11.7)	43.3 (19.0)	<0.001*
	Rarely	122 (24.2)	46.5 (17.1)	
	sometimes	148 (29.4)	51.8 (15.3)	
	Often	106 (21.0)	58.6 (16.6)	
	**Always**	**69 (13.7)**	**71.8 (16.7)**	

9. My bedtime varies a lot because of my shifts	Never	103 (20.4)	43.9 (19.3)	<0.001*
	Rarely	88 (17.5)	48.0 (15.6)	
	sometimes	132 (26.2)	51.1 (14.8)	
	Often	83 (16.5)	59.7 (17.3)	
	**Always**	**98 (19.4)**	**67.3 (17.6)**	

10. Did your job hours increase during the pandemic?	No	295 (58.5)	50.4 (18.1)	<0.001*
	**Yes**	**209 (41.5)**	**58.3 (18.8)**	

1. Too little vacations/inability to get requested vacation	Not at all	55 (10.9)	43.4 (18.4)	<0.001*
	Slightly	41 (8.1)	48.2 (19.6)	
	Somewhat	137 (27.2)	49.2 (16.4)	
	Moderately	136 (27.0)	55.0 (15.6)	
	**Extremely**	**135 (26.8)**	**62.7 (19.9)**	

2. Too many on-call shifts	Not at all	142 (28.2)	46.5 (17.5)	<0.001*
	Slightly	87 (17.3)	50.5 (19.7)	
	Somewhat	119 (23.6)	52.8 (15.8)	
	Moderately	92 (18.3)	57.8 (16.2)	
	**Extremely**	**64 (12.7)**	**69.6 (18.4)**	

3. Too many evening shifts	Not at all	171 (33.9)	47.4 (19.0)	<0.001*
	Slightly	70 (13.9)	51.0 (16.4)	
	Somewhat	119 (23.6)	52.5 (15.8)	
	Moderately	71 (14.1)	58.2 (18.1)	
	**Extremely**	**73 (14.5)**	**68.3 (16.9)**	

4. Too many weekend shifts	Not at all	136 (27.0)	44.7 (17.9)	<0.001*
	Slightly	59 (11.7)	50.1 (15.1)	
	Somewhat	98 (19.4)	50.8 (17.0)	
	Moderately	93 (18.5)	52.9 (14.1)	
	**Extremely**	**118 (23.4)**	**68.8 (17.2)**	

* According to significant value of ≤0.05; **Bold** indicates highest burnout score; SD: standard deviation.

### 3.3 Internal consistency of the surveys

Although the used surveys (burnout and COVID-19) were validated, internal consistency was assessed using Cronbach’s alpha test (Cronbach’s alpha for the burnout survey, 0.930; Cronbach’s alpha for the COVID-19 survey, 0.830).

### 3.4 Multiple linear regression analysis of factors affecting burnout

Age was a factor that predicted burnout, with younger age being associated with higher burnout levels [β =  − 0.333, 95% confidence interval (CI) =  − 0.534 to − 0.131, p < 0.001]. Another factor was gender, with female gender being associated with higher burnout levels (β = 3.815, 95% CI = 0.543–7.088, p = 0.022). In addition, pharmacy-related occupation was a factor affecting burnout (β =  − 1.243, 95% CI =  − 2.309 to − 0.176, p < 0.022). Adjusted and unadjusted demographic and job-related factors and the resulting β, 95% CI, and p values are presented in Table 3. In the COVID-19 survey, all questions with greater agreement predicted a higher burnout score. The question about feeling burnout during COVID-19 showed greater agreement and predicted a higher burnout score (unadjusted β = 8.336, 95% CI = 7.082–9.589, p < 0.001). Adjusted and unadjusted COVID-19 survey data and the resulting β, 95% CI, and p values are presented in Table 4.

**Table 3:** Adjusted and unadjusted multiple linear regression of factors affecting burnout score with resulting beta, p value and 95% CI.

**Items**	**Reference group**	**Unadjusted**			**Adjusted to age and gender**		
		Beta	Sig	95% CI (Lower-Upper)	Beta	Sig	95% CI (Lower – Upper)
Age (years)	None	−0.333	0.001*	−0.534 - −0.131			
Gender	Females to Males	3.815	0.022*	0.543–7.088			
Pharmacy related occupation	Higher categories to Pharmacy technician	−1.243	0.022*	−2.309 - −0.176	−1.071	0.056	−2.170–0.028
Highest academic degree	Higher categories to Diploma	−0.511	0.201	−1.295–0.273	−0.284	0.491	−1.093–0.525
Marital	Married to Single	−1.805	0.290	−5.152–1.543	1.987	0.324	−1.968–5.942
Nationality	Non-Saudi to Saudi	−2.147	0.420	−7.374–3.080	−0.042	0.988	−5.367–5.283
Smoking	Yes, to No	3.633	0.126	−1.026–8.292	5.844	0.017*	1.030–10.657
Years of experience	Higher categories to < 1 year	−1.222	0.052	−2.453–0.009	0.862	0.400	−1.148–2.873
Weekly working hours before pandemic	More hours to 21–40 h	0.758	0.563	−1.815–3.331	1.290	0.323	−1.273–3.853
Weekly working hours after the pandemic (in the last two months)	More hours to 21–40 h	1.439	0.137	−0.460–3.338	1.960	0.043*	0.065–3.855
Number of patients seen per day	None	0.074	0.841	−0.647–0.795	0.314	0.392	−0.406–1.035
Number of beds in the hospital	Other categories to Non-Available	−0.070	0.880	−0.979–0.839	0.027	0.955	−0.919–0.973

* According to significant value of ≤0.05; CI: confidence intervals.

**Table 4:** Adjusted and unadjusted multiple linear regression of factors affecting burnout score with resulting beta, p value and 95% CI.

**Items**	**Reference group**	**Unadjusted**			**Adjusted to age and gender**		
		Beta	Sig	95% CI (Lower – Upper)	Beta	Sig	95% CI (Lower – Upper)
1. I feel more burnout now as compared to before the crisis of COVID	Strongly disagree	8.336	<0.001*	7.082–9.589	8.291	<0.001*	7.052–9.530
2. I am worried about becoming infected	Strongly disagree	6.419	<0.001*	5.084–7.754	6.457	<0.001*	5.139–7.775
3. I am worried about my family becoming infected	Strongly disagree	6.221	<0.001*	4.455–7.988	6.286	<0.001*	4.545–8.027
4. I am worried about this going for too long	Strongly disagree	5.829	<0.001*	4.277–7.380	6.012	<0.001*	4.477–7.547
5. My workplace shows a Supportive culture	Never	−3.004	<0.001*	−4.375 - −1.633	−2.746	<0.001*	−4.113 - −1.380
6. I get too little sleep at night	Never	7.724	<0.001*	6.407–9.041	7.837	<0.001*	6.539–9.135
7. I have insomnia	Never	7.663	<0.001*	6.554–8.772	7.508	<0.001*	6.404–8.612
8. I wake up often during the night	Never	6.936	<0.001*	5.724–8.149	6.879	<0.001*	5.675–8.082
9. My bedtime varies a lot because of my shifts	Never	5.834	<0.001*	4.768–6.899	5.760	<0.001*	4.667–6.852
10. Did your job hours increase during the pandemic	No	7.927	<0.001*	4.664–11.190	8.258	<0.001*	5.016–11.501
1. Too little vacations/inability to get requested vacation	Not at all	4.904	<0.001*	3.680–6.129	4.839	<0.001*	3.623–6.055
2. Too many on-call shifts	Not at all	4.921	<0.001*	3.810–6.033	4.808	<0.001*	3.693–5.923
3. Too many evening shifts	Not at all	4.595	<0.001*	3.525–5.665	4.479	<0.001*	3.391–5.566
4. Too many weekend shifts	Not at all	5.272	<0.001*	4.296–6.247	5.151	<0.001*	4.164–6.139

* According to significant value of ≤0.05; CI: confidence intervals.

### 3.5 Thematic analysis of factors associated with burnout

Around 36 (7%) respondents answered to the open questions and attributed burnout to several factors, such as their work, the management and administration, patients, personal issues, and COVID-19 (Appendix A). Factors that were related to work, such as poor administration and management, lack of support from leadership, increased working hours, shortage of staff, too many (night/evening) shifts, and increased workload, were the main reasons for burnout in their opinion.

The respondents commonly mentioned blame culture and a lack of appreciation because their mistakes were amplified and there was no recognition of their efforts.

They also mentioned factors related to meeting patients and communicating with them.

Some respondents shared their personal struggles and feelings, including family stress.

The respondents also described COVID-19 as a factor associated with burnout because of the increased workload and the fear of getting infected and being isolated from their families.

## 4 Discussion

**Fig. 4:**
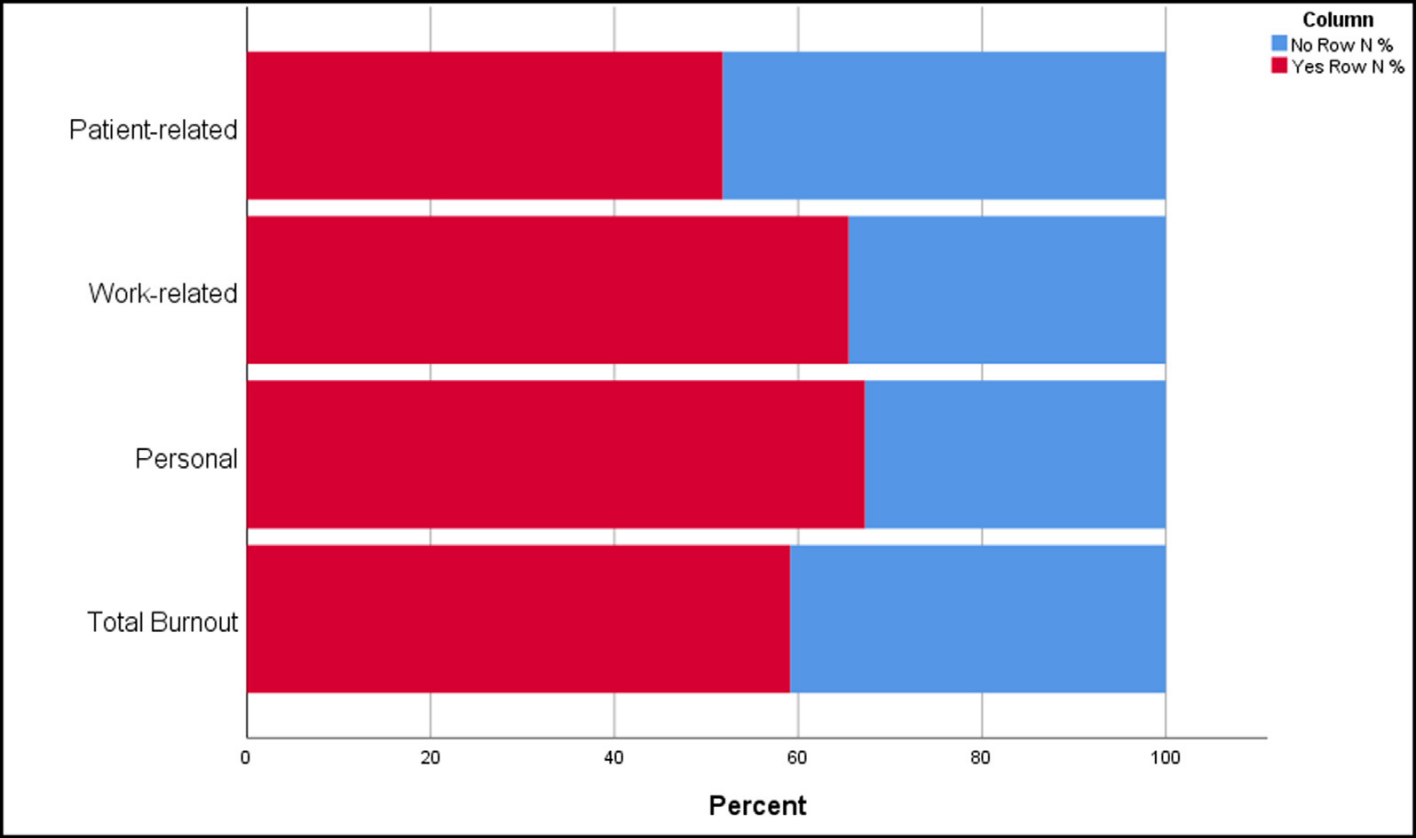
Types of burnout with precent of predominant type.

In the present study, around 60% of pharmacists were found to have clinically relevant burnout levels. Community pharmacists showed the highest burnout level among the study participants. Most respondents had higher scores in the personal part of the CBI (Fig. 4). Multiple studies have reported high burnout levels among healthcare professionals including pharmacists during the COVID-19 pandemic (Barello et al., 2020, Khasne et al., 2020, Matsuo et al., 2020, Morgantini et al., 2020). Many factors were identified to be associated with high burnout levels among pharmacists in the present study; these included age, gender, COVID-19, practice site, working culture, years of experience, and sleeping disturbances.

Age was found to be one of the risk factors in the present study, with an inverse relationship being noted between age and burnout levels. In other words, pharmacists with a younger age had higher burnout levels. In addition, having lesser years of experience was associated with higher burnout levels. Pharmacists who had 6–10 years of experience had high burnout levels; this also confirms our finding related to age, as pharmacists with lesser years of experience were mostly younger in age. Research on pharmacists and other healthcare providers has revealed that age and years of experience are factors related to burnout (Barello et al., 2020); in particular, younger age and lesser years of experience are associated with higher burnout levels (Alharbi et al., et al., 2020, Dugani et al., 2018, Matsuo et al., 2020). These findings can be explained by the fact that younger pharmacists have lesser work experience and are therefore likely to experience a higher emotional burden when performing their duties than senior pharmacists (Protano et al., 2019). Moreover, younger pharmacists are less likely to be involved in decision-making at their workplace (Rothmann and Malan, 2011), which can also intensify feelings of burnout, as younger generations often have higher job prediction (Alharbi et al., 2020). In contrast, in another study, older pharmacists with more years of experience were found to be at a higher risk of burnout. The risk of burnout also showed a direct correlation with job seniority. This is because senior pharmacists likely have higher fatigue and responsibilities and are involved for more years in dealing with patients (Protano et al., 2019). However, a study on physicians during the COVID-19 pandemic revealed that the median estimates of total burnout levels were not significantly related to age (Dinibutun, 2020).

Female gender was found to be another factor contributing to high burnout levels. Multiple studies on burnout among healthcare providers and pharmacists before and during the COVID-19 pandemic have reported higher burnout levels among female participants and considered female gender to be a risk factor (Barello et al., 2020, Khasne et al., 2020, Lange et al., 2020, Matsuo et al., 2020, Rubin et al., 2021, Zhou et al., 2020). This can be attributed to gender-specific challenges, including restricted childbearing years, being the primary caregiver for their baby, having fewer chances for promotion to leadership positions, and being paid unequally (Robinson, 2003). This finding can also be attributed to the dual role played by women at their home and at their workplace in the healthcare sector (Khasne et al., 2020). However, some studies have found that gender does not affect burnout levels (Di Monte et al., 2020, (Dinibutun, 2020).

Of all the respondents, community pharmacists had the highest burnout scores in the present study. A study among community pharmacists during the COVID-19 pandemic reported the presence of psychological disturbances, including elevated burnout symptoms (Lange et al., 2020).

The factors contributing to burnout in the present study included younger age, lesser years of experience, male gender, non-Saudi nationality, higher numbers of customers, and increased working hours.

A previous study found no association between gender and burnout levels among community pharmacists (Protano et al., 2019). In contrast, in another study, female community pharmacists were found to have higher burnout levels than male ones (Lange et al., 2020). This might be because community pharmacists in Saudi Arabia are mostly male.

Burnout levels of community pharmacists were associated with several comorbidities, including anxiety, depression, alcohol abuse, and the consumption of health resources (Balayssac et al., 2017). Community pharmacy is a complex system with several interconnected variables that impact patient safety (Al Juffali et al., 2019a), and medication safety priorities affecting community pharmacists have been established (Al Juffali et al., 2019b).

As community pharmacists are healthcare workers with the most accessibility to the public, they have a significant role to play in the COVID-19 pandemic response (Ahmad et al., 2020). Community pharmacists have shown readiness to play a supportive role by not only fighting the COVID-19 pandemic in the pharmacy but also helping to report individuals with suspected COVID-19; their multiple functions decrease the amount of unnecessary visits to hospitals, where the risk of COVID-19 is very high (Ahmad et al., 2020, Alshahrani, 2020). Community pharmacists often serve as a unique outlet for supplying patients with safety products, such as masks. Thus, at the beginning of the COVID-19, the interruption of surgical mask supply resulted in worries about responding to the high patient demand (Lange et al., 2020). Another factor related to community pharmacist is the type of pharmacy they work in (independent vs chain pharmacy), a higher level of stress is associated with pharmacist working in chain pharmacist (Jovičić-Bata et al., 2021). As the majority of community pharmacist in the present study are working in chain pharmacies. Pharmacists who are working in chain pharmacies have higher stress mainly because of larger number of prescriptions, time constraint, overtime (Jacobs et al., 2014).

Supportive work culture was another factor associated with higher burnout scores in the present study. The work environment has also been reported to play a role in burnout (Zhao et al., 2020) and to be a factor contributing to burnout. Hence, the importance of leadership in fostering a healthy work environment has been emphasized (Mudallal et al., 2017). A systematic review including meta-analysis of the work environment and burnout symptoms confirmed that the development of burnout syndrome is influenced by structural factors related to the work environment, including a non-supportive workplace (Aronsson et al., 2017). A global study assessing burnout during the COVID-19 pandemic has suggested that the actions of healthcare organizations and other governmental and non-governmental partners directed at potentially modifiable causes could reduce existing and future burnout among healthcare providers; these actions include the provision of additional preparation, organizational support, support for families of healthcare providers, and mental health services (Morgantini et al., 2020).

In the present study, COVID-19 was found to be strongly related to burnout because of multiple contributing factors, including fears of getting infected or infecting family members and of the pandemic continuing for too long. In addition, most participants felt that they were more burned out during the pandemic than before the pandemic. This attitude has also been detected in usual ward oncology physicians and nurses during the COVID-19 pandemic (Wu et al., 2020). As found in a study conducted in Serbia which found higher levels of stress were associated with pharmacist concerned for their family health (Jovičić-Bata et al., 2021).

The fear of infection was one of the factors associated with high burnout levels among Saudi pharmacists in the present study. In accordance with these findings, previous studies have also revealed that the fear of being infected or infecting a family member is a factor related to burnout (Algunmeeyn et al., 2020, Khasne et al., 2020, Zerbini et al., et al., 2020).

According to the WHO, burnout can have major effects on healthcare workers’ health by causing anxiety, irritability, mood swings, and depression (Aiken, 2002, Gundersen, 2001, Parker and Kulik, 1995, Shanafelt et al., 2002, Trufelli et al., 2008). Furthermore, burnout affects physical health by causing multiple aches and pains, indigestion, and cardiovascular risks, among others (Dyrbye et al., 2014, Eckleberry-Hunt et al., 2009, Maslach and Leiter, 2008, Salvagioni et al., 2017). Burnout has also been linked to increased job turnover and decreased productivity. In the healthcare sector, burnout not only affects an individual healthcare worker but also affects the quality of services provided to the patient and consequently affects patient outcomes (Dyrbye et al., 2014, Eckleberry-Hunt et al., 2009, Maslach and Leiter, 2008, Salvagioni et al., 2017). In a systematic review, most studies reviewed have been found that moderate to high burnout levels associated with poor patient safety outcomes, e.g., medical errors (Hall et al., 2016). Unfavorable outcomes, patient frustration, and increased patient and family problems are also correlated with higher burnout levels (Garcia et al., 2019). Another meta-analysis revealed that the burnout level of caregivers has strong unfavorable associations with perceived efficiency (including patient satisfaction), quality indices, and safety expectations (Salyers et al., 2017).

The COVID-19 pandemic has increased burnout levels because of the increased workload; increased emotional distress; decreased job control; increased infection rates; and uncertainty of the timeline of the disease, its treatment, and its complications. The emotional distress arising from the fear of getting infected or infecting family members, the change in work environment, and the increase in workload intensifies the effect of burnout on pharmacists (Dimitriu et al., 2020).

Different studies have provided several suggestions and recommendations to resolve this.

Interventions have revealed that organizational change may have a more significant impact, and several groups have outlined organization-focused strategies to improve well-being in the workplace (Dimitriu et al., 2020).

At the hospital level, the local agency has been suggested to play a far more critical role in reducing the level of stress and the incidence of burnout syndrome (Dimitriu et al., 2020). The presence of consistent procedures for all potential circumstances, practical preparation of the staff for security precautions, and sufficient use of protective equipment are measures to maintain a state of trust and power that can certainly lower the amount of stress (Dimitriu et al., 2020). Another study has revealed that micropractices can help prevent burnout; however, individuals who already have burnout should seek health support (Fessell and Cherniss, 2020). Moreover, one study has recommended telemedicine as a solution to maintain healthcare providers’ well-being (Moazzami et al., 2020).

A study on the psychological impact of COVID-19 has suggested that it is critical to develop mental health organizations to prepare for future pandemics with branches in many countries, including individual healthcare facilities; to implement mental health services; and to organize awareness campaigns at both personal and community levels (Dubey et al., 2020).

A limitation of the present study was that similar numbers of participants could not be achieved in each group, resulting in a higher number of pharmacists in one group than in another. The number of pharmacists in each category was different. For instance, the number of technicians was very low. Moreover, no previous studies conducted in Saudi Arabia were available for comparison; the only available study was limited to National Guard Hospital pharmacists.

Because of the very busy working situation during the COVID-19 pandemic, some very targeted populations may not have responded. In addition, pharmacists who were feeling more pessimistic or had higher burnout could have shown more inclination to respond to the survey.

Despite these limitations, the present study has major strengths. It is the first study in Saudi Arabia to assess burnout among pharmacists at a national level and includes both hospital and community pharmacists. The sample size is considered acceptable (see Table 5).

**Table 5:** Factors affecting community pharmacy.

	**B**	**Sig.**	**Exp (B)**	**95% confidence intervals**
Age (years)	−0.070	<0.001*	0.932	0.901–0.964
Gender (being a male)	0.535	0.011*	1.708	1.128–2.585
Marital (being single)	0.293	0.178	1.341	0.875–2.054
Nationality (being Saudi)	−1.183	<0.001*	0.306	0.173–0.543
Smoking (not smoking)	−0.536	0.051	0.585	0.341–1.002
Years of experience (years)	−0.390	<0.001*	0.677	0.566–0.809
40–60 Working hours	−1.190	0.151	0.304	0.060–1.546
61–80 Working hours	−1.275	0.126	0.280	0.055–1.432
>80 Working hours	−0.368	0.691	0.692	0.113–4.239
Number of customers per day	0.076	0.087	1.079	0.989–1.177

To the best of our knowledge, this is the first study on the impact of the COVID-19 pandemic on the burnout of pharmacists. The results represent pharmacy staff burnout during the peak of the pandemic in Saudi Arabia, between June 19 and September 30, 2020 when the cases of COVID-19 were increasing and there was uncertinatity about the vaccine.

## 5 Conclusions

The present study revealed that Pharmacy staff in Saudi Arabia experience high levels of burnout. The major risk factors associated with burnout included younger age, female gender, lesser years of experience, a lack of supportive work culture, and sleep disturbances. The COVID-19 situation and the consequent fears of being infected or infecting family members, increased job hours, and worries about the pandemic going on for too long were also strongly related to high burnout levels. Community pharmacists had higher burnout scores than the other groups of pharmacists. Solutions such as micropractices and telemedicine should be considered by the administration and management. More research to identify other solutions is also needed in order to overcome the increased prevalence of burnout syndrome during the COVID-19 pandemic.

## 6 Consent for publication

Electronic informed consent was obtained from the study participants.

## 7 Data availability (where applicable)

The datasets used and/or analyzed in the present study are available from the corresponding author upon reasonable request.

## 8 Funding.

None.

## 9 Authors’ contributions

All authors contributed extensively to the work presented in this paper, drafted or substantially revised the article, reviewed the final manuscript, agree to take responsibility for the content of the article, have approved the manuscript, and agree with its submission to Saudi Pharmaceutical journal.

## Declaration of Competing Interest

The authors declare that they have no known competing financial interests or personal relationships that could have appeared to influence the work reported in this paper.

## Figures and Tables

**Table:** 

**Themes**		**Quote**					
**Work -related**	**Shortage of staff-nightshifts**	“My burn-out isn’t because of my work with patients, I actually love what I do. My exaction and tiredness is because of night shifts and evening shifts that we have on a monthly bases and for almost a week, we are short on staff compared to the work need to be done, we have nurses coming and going to the pharmacy, without knowing if they had the COVID-19 or not, we had a large number of staff who got infected while covering in the pharmacy, our problem is administrative not COVID related, and it has been like that for the past 4 years.” **Participant-1**					
	**Blame culture**	“Emphasis of blame culture whenever a mistake is done, where if work is going smoothly not even a word of thanks or encouragement is told. ” **Participant-2**					
	**Working environment**	“due to the working environment and the working hours for the salary I get is really not satisfying” **Participant-3**	**“In health centers, there is a lot of neglect of pharmacists and pharmacist technicians in terms of training and continuous courses for long periods. Thank you and good luck.” **Participant-4**	“Environment of work make difference” **Participant-5**			
	**Working hours**	“I work in a community pharmacy 8 h a day with only 2 days off in 1 month, 11 working hours on Fridays no family no friends no security, I just wanna die, pray for me:(” **Participant-6**					
	**Vacation**	“We need vacation or at least days off” **Participant-7**					
	**Management**	“It's the stress caused by poor management and lack of support from leadership that causing the most harm. It's hard to work for an unappreciative manager.” **Participant-8**	“1. Heads, directors, and supervisors concern on presence rather than productivity! 2. Being in an environment where everything/updates/privileges seems secretive! ” **Participant-9**				
	**Lack of appreciation**	“To my opinion; up until now stakeholders don’t get the pharmacist job seriously. They don’t know or may be ignore how much time we need in order to process orders. It is like if they just want the job to get done and that’s it. Most of them want us to follow the prescribers orders as they are. Those who know how pharmacy major works know that we take time to make sure what is prescribed ALWAYS needs verification in terms of the dose/indication/frequency as well as duration. Time needed to process an order is always fallen out of the count. It could be because they lack sense of applying the right job’s perspective. Although I have been there, but what makes the feeling of burn out goes away is that I’m doing what I can to contribute to the patient safety, performance enhancement, and eventually the country’s mission and vision” **Participant-10**	“Lack of appreciation of the work by the line manager.” **Participant-11**“Culture support specially departmental one. Respect the employee effort. Say Thank you for all staff. ” **Participant-12**	“No support from the administration at all and no benefits, ” **Participant-13**	**“It is true that nurses and doctors are carrying the most effort, but the support for them is great from their departments and the health pyramid. While pharmacists are the unknown soldier in this episode, unfortunately, their administration's support for them is weak. On the contrary, some of them faced injustice and some managers reach the point of forcing the employee to dismiss him from work” **Participant-14**	“Take care you have to be patient especially with people who doesn't respect your career” **Participant-15**	**“Can you share this research so an infection allowance would be approved for pharmacists, like other specialties” **Participant-16**

**Job satisfaction**		“Working as usual, Instead, I’m feeling very valuable when working during this crisis.” **Participant-17**	“Everything is fine, Thanks for caring about our mental health” **Participant-18**	“I don’t feel like that at all” **Participant-19**			
**Patient related**		“I have been working in this field for 8 years and I faced a lot with people it's very tough to face all of complains and patient problems” **Participant-15**	“sometimes when patients do not follow the medical instructions or taking safety precautions makes me uncomfortable.” **Participant-20**				

**Personal**	**Personal struggles**	“I feel very exhausted at this job, every day I think of submitting a resignation, and I will be relieved because I have nothing to lose” **Participant-3**	“feeling like spinning in circles, my work is wasted, does not benefit the patient nor the hospital, always something missing, lack of understanding, support and inclusion in work designs.” **Participant-21**	“Waiting for the moment to change my work.” **Participant-22**	“I am sorry for all of these bad experience and I know that isn't a scale related to what happened last years” **Participant-15**		
	**Blue, sadness**	“I work in a community pharmacy 8 h a day with only 2 days off in 1 month, 11 working hours on Fridays no family no friends no security, I just wanna die, pray for me:(” **Participant-6**	“Sometimes I wish I can live alone” **Participant-23**				
	**family issues**	“I am thinking to quit my job. since I had a new baby” **Participant-24**					

**COVID-related**	**work related**	“It is tiring to feel so much pressure from patients, work place, your supervisor and Corona infection.. work load becomes heavier even work hours is less, pharmacist even thought hard working we are we are not a seen as important part of the team even we are dealing with patient medication leading to dealing with their life no appreciation seen ..” **Participant-25**	“Working in a hospital especially through pandemic period is very exhausting and causing illness and suffering to majority of the staff” **Participant-26**	“Hope COVID will ends soon because I am burned out already” **Participant-27**	**“I see that pharmacists are the most consumed and affected employees” **Participant-14**		
	**fear**	“There is a great psychological effect on the pharmacists being coming from a place of the first degree of the infection environment.” **Participant-28**					
	**patients**	“Community awareness of the danger of disease” **Participant-29**	“it is sometimes difficult to answer people regarding span of COVID-19.” **Participant-30**				

**Others**		**“God knows all these bad situations and tiredness that we are through and he would reward us with the best, rely on god and do not be afraid, whenever you feel frustrated keep saying the prayers it will comfort you and make you feel better you are heroes and you can do it” **Participant-31**	“If you ever feel like negativity you have to go back to Allah and everything will be alright” **Participant-32**	“Be relaxing any time and anywhere” **Participant-33**	“We still stand until corona finish” **Participant-34**	“If any one he has exhaustion , and he didn’t found any solution, should to increase his relationship with Allah, to take help, thank you for this survey □” **Participant-35**	“Lack of supervision during my training” **Participant-36**

## Appendix A Qualitative theme of the open-ended question

** This is an English translation from Arabic, the original respond was written in Arabic language.
